# A role for Shugoshin in human cilia?

**DOI:** 10.17912/micropub.biology.001013

**Published:** 2023-12-12

**Authors:** Rachel Reed, Jennifer N.K. Nyarko, Darrell D. Mousseau, Carlos Egydio de Carvalho

**Affiliations:** 1 Biology, Department of Biology, University of Saskatchewan; 2 Department of Psychiatry & Physiology, University of Saskatchewan; 3 Department of Biology, Department of Biology, University of Saskatchewan

## Abstract

We have recently described a novel role for the conserved centromeric/kinetochore protein and cohesin protector, Shugoshin, in cilia of *C. elegans.* Worms are unusual in that the sole Shugoshin protein ( SGO-1 ) is dispensable for chromosome segregation but required for cilia function in fully differentiated sensory neurons. Depletion of *sgo-1* leads to an array of sensory defects observed in other cilia mutants with a compromised diffusion barrier. Accordingly, SGO-1 loads to the base of cilia in sensory neurons and can be observed occupying the transition zone, the critical ciliary domain that regulates trafficking in and out of ciliary compartments. Here we start to address a potential conserved role in cilia for vertebrate Shugoshin by asking whether human Shugoshin can: ^(1)^ localize to cilia and ^(2)^ rescue defects due to Shugoshin depletion in *C. elegans* . Our preliminary results suggest that human Shugoshin is detectable in the cilia base but show limited functional conservation when expressed in *C. elegans* sensory neurons.

**Figure 1. Human Shugoshin is observed in the base of cilia in human neuroblastoma cells and localizes to distal dendrites and cilia proper when ectopically expressed in C. elegans sensory neurons:**
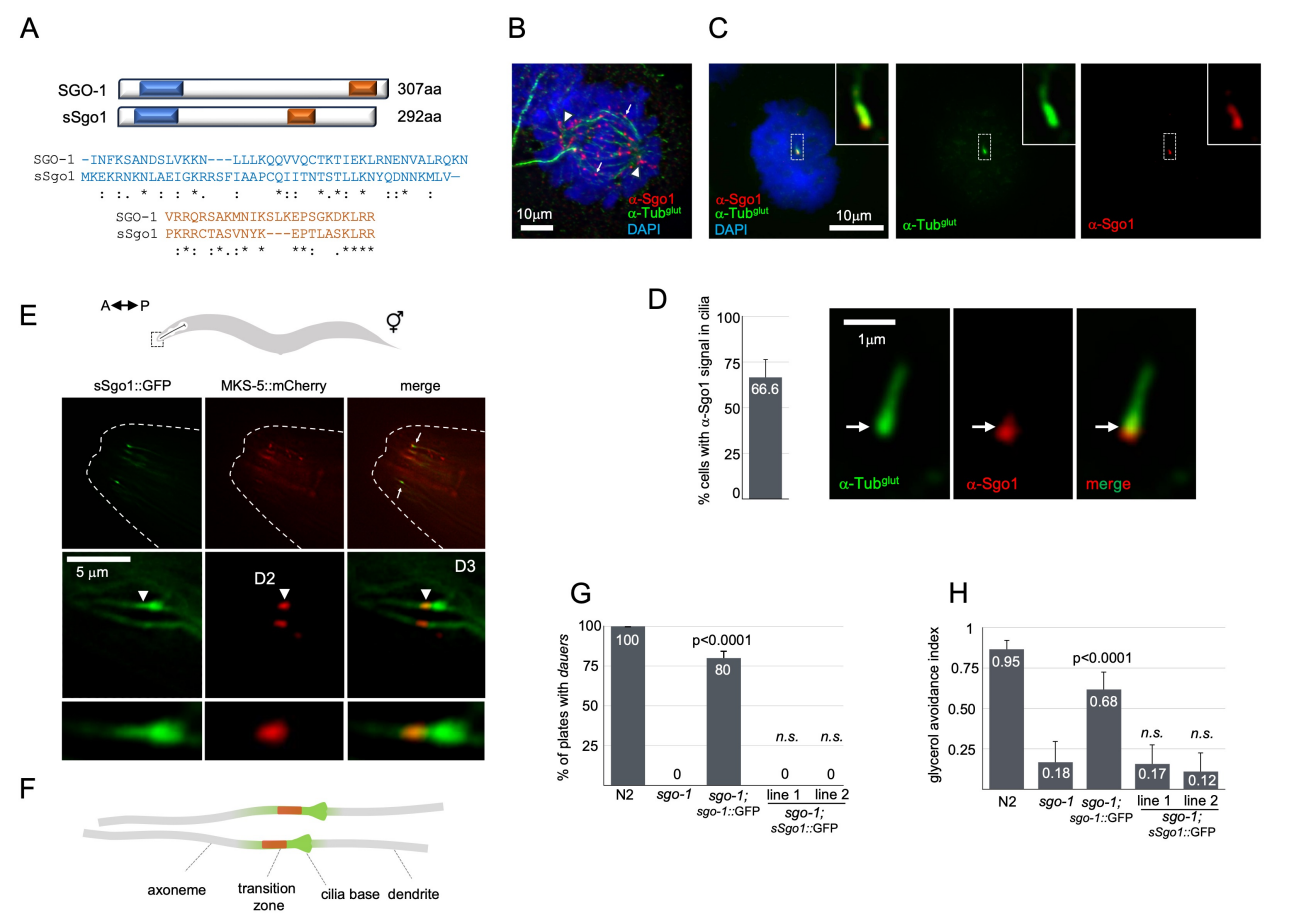
( **A** ) Diagram of the *C. elegans* SGO-1 ( C33H5.15 - NP 001294168.1) protein and the human sSgo1 isoform 1KL (Q5FBB7-3 transcript - NP 001186185.1). The N-terminus coiled-coil domain (IPR011516) is highlighted in blue and the C-terminus basic (Shugoshin) domain in brown (IPR011515). ClustalW alignments ( *https://www.genome.jp/tools-bin/clustalw* ) between human (Sgo1) and *C. elegans* Shugoshin ( SGO-1 ) proteins for the N and C termini domains are shown in the bottom - (*) indicates positions with a fully conserved residue; (:) indicates that one of the following 'strong' groups is fully conserved - STA NEQK NHQK NDEQ QHRK MILV MILF HY FYW; (.) indicates that one of the following 'weaker' groups is fully conserved - CSA ATV SAG STNK STPA SGND SNDEQK NDEQHK NEQHRK FVLIM HFY. ( **B** ) Representative human neuroblastoma SH-SY5Y cell in metaphase-anaphase transition immunostained with anti-Sgo1 (red) and anti-glutamylated tubulin (green) antibodies. The commercial antibody used in this study reveals the expected Sgo1 staining on mitotic chromosomes (centromeres) in the metaphase plate (arrows) and in the spindle poles (arrow heads). ( **C** ) Representative serum-starved, ciliated SH-SY5Y cell stained with the same antibodies as in (B). Insets of marked cilia regions (boxes in A) are shown in the top right corners. ( **D** ) Quantification of ciliated cells displaying co-localization of anti *-* Sgo1 and anti-Tubulin signals is shown (n=123). Representative zoomed in panels of a stained cilium is shown on the right. Arrows indicate the base of the single cilium per cell. ( **E** ) Targeted heterologous expression of a human sSgo1 cDNA ::GFP transgene in sensory neurons of hermaphrodites co-expressing the TZ marker MKS-5 ::mCherry. Top microscopy panels show zoomed out images of the nose region, corresponding anatomically to the boxed region marked in the hermaphrodite diagram (A-P, anterior/posterior axis) . Arrows point to the amphid region. Zoomed in images of amphid cilia is shown in the two bottom panels. Note that in addition to a proximal cilia localization, sSgo-1::GFP signal is detected crossing into the cilia proper and occupying the TZ region distally (arrow heads). ( **F** ) Cartoon of the amphid cilia. sSgo1 expression in *C. elegans* cilia does not functionally rescue dauer formation ( **G** ) nor the osmotic avoidance behavior defect ( **H** ) of *sgo-1 ( tm2443 )* mutant worms.

## Description

Originally identified in flies and yeast based on their essential role in ensuring timely chromosome separation in anaphase, Shugoshins show poor sequence homology across species (Watanabe, 2005; Gutiérrez-Caballero et al., 2012). Most Shugoshin proteins have been identified bioinformatically based on the presence of a coiled coil domain in the N-terminus and an arginine-rich, basic domain in the C-terminus. (Kitajima et al., 2004; Rabitsch et al., 2004; **Figure 1A** ). Along with weak amino acid conservation, Shugoshins show several species-specific protein partners and specialized functions in different cell division contexts, arguing against a direct orthology, and complicating the understanding of the evolution of this protein family (Gutiérrez-Caballero et al., 2012; Grishaeva et al, 2016). Unifying among these functions, however, are the sites of action of Shugoshins: the kinetochore, spindle, centrosome and primary cilium, all of which are microtubule-based cellular structures.

The *C. elegans* genome encodes a single Shugoshin homolog, SGO-1 , that localizes to chromosomes in mitotic and meiotic cells, and to cilia in the adult sensory organs (de Carvalho 2008; Bohr 2016, Reed et al., 2023) . Surprisingly, SGO-1-depleted worms are viable and fertile but show behavioural defects consistent with cilia dysfunction. We asked whether the cilia function of SGO-1 in worms might be a conserved feature in humans, in which two Shugoshin paralogs exist, Sgo1 (Sgol1) and Sgo2 (Sgol2). We chose to concentrate on Sgo1 as the candidate for a potential function in cilia for three reasons. First, sSgo1, a shorter splice variant of human Sgo1, functions outside of the nucleus where it regulates centriolar cohesion during mitosis (Wang et al., 2008) . Second, immunostaining evidence in mouse supports the presence of Sgo1 isoforms in the cytoplasm of post-developmental retinal ganglion neurons, a ciliated cell type (Song et al., 2017a; Song et al., 2017b). Third, a substitution in the N-terminal domain of Sgo1 (K23E) disrupts its ability to directly interact with the pacemaker ion channel HCN4 in the membrane of cardiomyocytes of patients with Chronic Atrial and Intestinal Dysrhythmia Syndrome (CAID) (Liu et al., 2021) . Thus, Sgo1 has likely evolved different extra-nuclear activities. Of note, sSgo1 (292aa) lacks 268 residues encoded by exon 6, but retains both conserved Shugoshin domains and is of similar size as *C. elegans* SGO-1 (307aa; C33H5.15 a.1) ( **Figure 1A** ). For this analysis we used human SH-SY5Y neuronal-like cells that were induced to enter ciliogenesis by serum starvation. The presence of cilia was confirmed with an anti-polyglutamylation antibody that marks axonemal tubulin (Tub ^glut^ ). To detect Sgo1, we used an affinity purified polyclonal anti-Sgo1 antibody that specifically recognizes both human Sgo1 isoforms (Agircan & Schiebel, 2014) . We first validated that this antibody could detect Sgo1 in SH-SY5Y mitotic figures. Immunostaining of SH-SY5Y cells in anaphase showed the expected foci-like staining attributed to centromeres ( **Figure 1B** ). In interphasic cells, we did not detect positive anti-Sgo1 staining on chromosomes, but rather detected a dispersed signal within the cytoplasm. A similar signal distribution for Sgo1 has been described elsewhere in mouse brain sections using monoclonal anti-mouse Sgo1 antibodies (Song et al., 2017a). We found that among SH-SY5Y cells with anti-Tub ^glu^ -marked cilia, around 65% showed strong anti-Sgo1 signal co-localizing with anti-Tub ^glut^ ( **Figure 1C, D** ). The Sgo1 signal was strongest at the base of the cilium, in a region consistent with the basal body, but also extended distally into the ciliary shaft. In the remaining anti-Tub ^glu^ positive cells, no detectable anti-Sgo1 signal was observed in or around cilia. The localization of Sgo1 in SH-SY5Y cells is reminiscent of SGO-1 localization in worms, which is observed primarily in the cilia base and transition zone of sensory cilia (Reed et al., 2023) . Definitive proof of a cilia localization for Sgo1 will require, however, the investigation of *sgo1* -depleted cells.

Considering its low homology with *C. elegans* SGO-1 , the presence of Sgo1 in cilia of neuroblastoma cells was surprising and hinted at potential functional conservation in this organelle. To gain insight into a possible shared ciliary function between human and *C. elegans* Shugoshin, we expressed sSgo1::GFP in *C. elegans* sensory neurons using a pan-cilia promoter *(Posm-5* ) and asked whether the human protein could localize to cilia in worms. In these heterologous overexpression lines, we detected sSgo1::GFP on sensory organs in the head of hermaphrodites ( **Figure 1C, D** ). While we cannot rule out the contribution of overexpression in the dendritic and periciliary sSgo1::GFP signal, we noticed that, as observed already with SGO-1 , sSgo1 is able to cross into the highly impermeable transition zone region, suggesting that the cilia localization of sSgo1::GFP is the product of active transport. It remains possible, however, that transport of sSgo1 into cilia is not biologically relevant in worms (Reed et al., 2023, inset panels in **Figure1 E** ). We reasoned that if sSgo1 has a conserved function in the cilia proper, it may be able to rescue sensory defects in *sgo-1* mutant worms. To assess if sSgo1 is functionally redundant with SGO-1 in cilia, we expressed sSgo1::GFP in sensory neurons of *sgo-1 ( tm2443 )* worms and evaluated whether the human protein could rescue the sensory defects in these animals carrying a truncated protein product (Reed et al., 2023) . We found no evidence of this: while *sgo-1* ( *tm2443* ) worms can be partially rescued by a SGO-1 ::GFP transgene, mutant worms expressing sSgo1::GFP display similar levels of dauer formation and glycerol avoidance defects as *sgo-1* ( *tm2443* ) mutants ( **Figure 1G, H )** . The failure of a human Sgo-1 to rescue *sgo-1* sensory defects in worms is not unexpected, in view of how Shugoshin proteins have diverged both sequence-wise and functionally in different species. However, these results do not rule out a potential ciliary function of Sgo1 in human cells, a possibility that was not investigated here.

Several integral kinetochore components of the Knl1/Mis12/Ndc80 (KMN) network and transient kinetochore proteins such as Aurora kinases that mediate microtubule attachment and chromosome segregation have been recently implicated in the development of cilia-containing sensory organs during *C. elegans* embryonic development (Cheerambathur et al., 2019; Bai et al., 2020) . Together with the extra-nuclear localization of Shugoshin proteins in neuronal cells, these findings reinforce the perception that protein modules controlling the build up and disassembly of transitory microtubule-based structures during cell division and ciliogenesesis have been extensively co-opted during evolution.

In conclusion, our preliminary results raised the intriguing possibility that vertebrate Shugoshin may access and perhaps regulate primary cilia function. Further experiments looking at expression of *sgo1* isoforms in human cells and assessing cilia defects upon *sgo1* depletion will be necessary to confirm these findings and probe into any functional implication. Nevertheless, a conserved role of human Shugoshin in cilia will invite a revision of the current focus in understanding pathologies recently linked to Shugoshin mutations in fully differentiated cells, such as CAID and Alzheimer’s disease, from exclusively cohesinopathic in origin to a possible ciliopathic perspective (Rao et al., 2018) .

## Methods

**Culture and immunostaining of SH-SY5Y cells**

2x10 ^5^ SH-SY5Y cells were seeded on poly-D-lysine coated coverslips and allowed to establish in DMEM/F12 (1:1) (HEPES) + 10% FBS for 24 hours. 48 hours of serum deprivation (0.5% FBS) was used to induce ciliogenesis. Cells were fixed in 4% formaldehyde solution at room temperature for 20 minutes followed by incubation in 0.1 M glycine in PBS for 5 minutes. After three washes in PBS, cells were permeabilized with 0.5% Triton X-100/PBS for 30 minutes at room temperature, washed once in PBS for 5 minutes and blocked under gentle agitation with a 5% Normal Donkey Serum + 1% BSA solution. Primary antibody incubation was performed in 1% Normal Donkey Serum + 1% BSA overnight at 4°C. After washing three times with 0.05% Triton X-100 / PBS solution, cells were exposed to the secondary antibody for 1 hour at room temperature before mounting using ProLong™ Glass Antifade Mountant (Invitrogen; P36980). Primary antibodies used were: 1:500 rabbit anti-Sgo1 polyclonal antibody (Invitrogen; #PA5-30869; from now on referred as anti-Sgo1) and 1:1000 mouse anti-polyglutamylation modification monoclonal antibody (AdipoGen Life Sciences; AG-20B-0020-C100; anti-Tub ^glut^ ). Secondary antibodies used were: anti-rabbit IgG Alexa Fluor Plus 594 (1:2,000; Invitrogen) and anti-mouse IgG Alexa Fluor Plus 488 (1:2,000; Invitrogen). Two rounds of staining were performed. As control for staining, mitotic figures with expected anti-Sgo1 signal on centromeres were confirmed on both experiments. Cells with anti-Tub ^glut^ signal were scored for presence or absence of anti-Sgo1 signal in cilia to calculate the prevalence of Sgo1 in cilia.

***C. elegans* strains maintenance**

*C. elegans* strains were cultured on Normal Growth Medium (NGM) plates at 20°C in accordance with standard conditions (Brenner, 1974) . Strains used in this study are listed in **Table 1** .

**Microscopy**

Cell and worm images were taken using a Delta Vision deconvolution system (GE). 3D projections of image stacks (0.2 μm optical sections) are shown. When necessary, image deconvolution was accomplished using Softworxs (GE). For imaging *C. elegans* , two-day old adult worms were used. Animals were mounted on 2% agarose pads with 25 mM of sodium azide in M9 buffer and imaged immediately.

**Glycerol avoidance**

The drop test method was used to quantify avoidance behavior to 3M glycerol (Hilliard et al., 2004) . In this test, a drop of glycerol solution is placed on the tail of a forward moving worm tracking on an agar surface. As the solution reaches the sensory organs in the head by capillarity, an immediate backward movement response follows. Failure to back within 4 seconds of entering in contact with the solution is scored as lack of avoidance. Tests were repeated for the same worms 10 times. 30 worms per genotype were tested for a total of 300 avoidance responses recorded. Avoidance indexes (AI) for each worm (the number of positive avoidance movements divided by the number of tests and AI averages) were calculated. Only worms that completed all 10 tests were considered for calculation.

**Dauer formation**

Resistance to SDS treatment was used to quantify dauer formation (Cassada et al., 1975) . Mixed worm populations derived from four L4 hermaphrodites per plate were grown on standard size (1 cm ^2^ ) OP50 lawns and monitored for starvation. Five days after food depletion, worms were collected and washed in M9, resuspended in 1 ml of 1% SDS and incubated with gentle shaking for 30 min. Surviving dauers were identified on petri dishes thrashing under a Nikon 745 stereoscope. Three experiments were carried out, each composed of 12 independent plates. Plates were scored for the present or absence of dauers. The % average of plates containing dauers in the three experiments is presented.

**Statistics**

Significance (p<0.05) was calculated using two-tailed t-tests with equal variance (MS Excel).

**Cloning of *Posm-5::sSgo1::GFP* and microinjection into gonads**

A *C. elegans* codon-optimized human *sSgo1* cDNA (876bp, see sequence below) was cloned downstream of the ciliated neuron specific promoter *Posm-5* (329bp) in pUC57 to make pCEC62. Subsequently the *Posm-5::sSgo1* insert was subcloned upstream to and in frame with the GFP cassette in pPD95.75 using SphI / KpnI, to make pCEC65 ( *Posm-5::sSgo1::GFP:: unc-54 UTR)* . All plasmids were confirmed by sequencing. Microinjection was performed using a Nikon inverted microscope equipped with a Narishige Micromanipulator. pCEC65 and *Parl-13:: mks-5 ::mCherry:: unc-54 UTR* plasmid were injected into N2 gonads to make CEC279 worms. *sgo-1 ( tm2443 ); Posm-5::sSgo1::GFP:: unc-54 UTR* rescue lines (CEC290 and CEC291) were made by injecting pCEC65 into *sgo-1 ( tm2443 )* worms. ccGFP (pCFJ68) was used as co-injection marker to generate these strains. A list of plasmids is provided in **Table 2** .

>sSgo1 codon optimized for *C. elegans* (no stop)

ATGGCTAAAGAAAGATGTCTTAAAAAATCTTTCCAAGATTCTCTTGAAGATATTAAAAAAAGAATGAAAGAAAAAAGAAATAAAAATCTTGCTGAAATTGGAAAAAGAAGATCTTTCATTGCTGCTCCATGTCAAATTATTACTAATACTTCTACTCTTCTTAAAAATTATCAAGATAATAATAAAATGCTTGTTCTTGCTCTTGAAAATGAAAAATCTAAAGTTAAAGAAGCTCAAGATATTATTCTTCAACTTAGAAAAGAATGTTATTATCTTACTTGTCAACTTTATGCTCTTAAAGGAAAACTTACTTCTCAACAAACTGTTGAACCAGCTCAAAATCAAGAAATTTGTTCTTCTGGAATGGATCCAAATTCTGATGATTCTTCTAGAAATCTTTTCGTTAAAGATCTTCCACAAATTCCACTTGAAGAAACTGAACTTCCAGGACAAGGAGAATCTTTCCAAATTGAAGCTACTCCACCAGAAACTCAACAATCTCCACATCTTTCTCTTAAAGATATTACTAATGTTTCTCTTTATCCAGTTGTTAAAATTAGAAGACTTTCTCTTTCTCCAAAAAAAAATAAAGCTTCTCCAGCTGTTGCTCTTCCAAAAAGAAGATGTACTGCTTCTGTTAATTATAAAGAACCAACTCTTGCTTCTAAACTTAGAAGAGGAGATCCATTCACTGATCTTTGTTTCCTTAATTCTCCAATTTTCAAACAAAAAAAAGATCTTAGAAGATCTAAAAAAAGAGCTCTTGAAGTTTCTCCAGCTAAAGAAGCTATTTTCATTCTTTATTATGTTAGAGAATTCGTTTCTAGATTCCCAGATTGTAGAAAATGTAAACTTGAAACTCATATTTGTCTTAGA

## Reagents

**Table 1. *C. elegans* strains**

**Table:** 

**strain**	**genotype**	**source**
N2	*Caenorhabditis elegans* wild type isolate	CGC
CV138	*sgo-1 ( tm2443 ) *	M. Colaiacovo
CEC279	* sasEx75 [Posm-5::sSgo1::GFP:: unc-54 UTR; Parl-13:: mks-5 ::mCherry::unc-54 UTR; ccGFP] *	This work
CEC290	*sgo-1 ( tm2344 )IV ; sasEx83 [Posm-5::sSgo1::GFP:: unc-54 UTR; ccGFP] line 1 *	This work
CEC291	*sgo-1 ( tm2344 )IV ; sasEx84 [Posm-5::sSgo1::GFP:: unc-54 UTR; ccGFP] line 2 *	This work
CEC239	*sgo-1 ( tm2443 )IV; sasEx52 [Posm-5:: sgo-1 ::GFP; Podr-1::DsRed] *	Reed et al., 2023

**Table 2. Plasmids**

**Table:** 

**plasmid**	**Genotype | Information**	**source**
pCFJ68	* Punc-122::GFP:: unc-54 3’UTR (ccGFP) | available at Addgene *	E. Jorgensen
--	* Parl-13:: mks-5 ::mCherry:: unc-54 UTR | marker for TZ *	Wei et al., 2013
pCEC62	*Posm-5::sSgo-1cDNA | human sSgo1 - codon-optimized for C. elegans*	This work
pCEC65	* Posm-5::sSgo-1cDNA::GFP:: unc-54 UTR | Cilia-driven sSgo-1::GFP translation reporter *	This work
